# Agreement between the activPAL accelerometer and direct observation during a series of gait and sit-to-stand tasks in people living with cervical dystonia

**DOI:** 10.3389/fneur.2024.1286447

**Published:** 2024-04-24

**Authors:** Irum Yaqoob, Silmara Gusso, Mark Simpson, Rebecca M. Meiring

**Affiliations:** ^1^Department of Exercise Sciences, Faculty of Science, The University of Auckland, Auckland, New Zealand; ^2^Department of Neurology, Auckland City Hospital, Auckland, New Zealand; ^3^School of Physiology, University of the Witwatersrand, Johannesburg, South Africa

**Keywords:** activPAL, accelerometer, agreement, cervical dystonia, physical activity, sedentary behaviour

## Abstract

**Background:**

Accelerometers are commonly used for the assessment of PA; however, these devices have not been validated in people with dystonia who experience movement limitations. To properly understand movement behaviors and deliver accurate exercise prescription in this population, the validity of these devices must be tested.

**Objective:**

This study aimed to validate step count and postural transitions detected by the activPAL accelerometer (AP) against direct observation (DO) during two functional assessments: the 30-s sit-to-stand (30STS) and 6-min usual-pace walk tests. Methods: A total of 11 participants with cervical dystonia (CD) (male/female *n* = 5/6; mean age = 61 years; BMI = 24 kg/m^2^) performed the 6-min usual pace walking and 30STS while wearing the activPAL. A trained observer counted steps and observed the number of sit-to-stands.

**Results:**

The average step count detected with AP and DO was 651.8 (218–758) and 654.5 (287–798) respectively. The average transitions detected were 11 (4–16) and 12 (4–17) respectively. Both methods showed good agreement and there was a statistically significant and strong correlation between the two methods, i.e., transitions (*r* = 0.983, *p* = 0.0001), and step counts (*r* = 0.9841, *p* = 0.0001).

**Conclusion:**

There is a good agreement between activPAL and direct observation for step counts and transitions between sitting and standing in people living with CD.

## 1 Introduction

Dystonia is an idiopathic disorder, the third most common among movement-related disorders (1). Cervical dystonia (CD) is an isolated form of dystonia affecting 10 in 10,000 people (2), comprising 40% of all dystonias (3). CD affects the neck, resulting in twisted, painful, and non-functional positions of the head (4). Physical function may be impaired in CD, due to the motor and non-motor symptoms (5) that disrupt balance in walking, increasing the risk of falls and (6, 7) resulting in avoidance of participation in physical activity (3). Motor impairments are linked to the pathological impairments affecting different brain areas in cervical dystonia. These areas may include the somato-sensory cortex (impairments causing disruption of movement and position sense), basal ganglia (leads to disruption in mechanism controlling the excessive movement), and cerebellum (results in disruption of balance and postural control) (6, 7). Sometimes visual impairment due to the lack of neck stability may also lead to the loss of balance (6).

There are currently no disease-specific physical activity (PA) guidelines for people with dystonia. As per general population guidelines (8), adults with dystonia should take part in at least 150 min of moderate-intensity physical activity per week (9). PA engagement should be encouraged not only to improve overall health but also to minimize secondary complications, including balance and gait deviations (6, 7, 10–12). Accurately measuring and understanding current PA levels in people with dystonia can assist health care professionals and researchers to develop effective health management plans aimed at promoting health and wellbeing through suitable physical activity engagement.

Several established methods are used to estimate levels of physical activity, each with their own benefits and limitations. Doubly-labelled water is considered the gold standard for estimating energy expenditure during activity (13, 14) but is an expensive and time-consuming method unsuitable for use in free-living settings (15, 16). Self-reported physical activity questionnaires are convenient for distribution in large sample settings, however they are associated with over reporting of physical activity and underreporting of sedentary behavior (SB) levels (17, 18). Accelerometers are an increasingly common way of estimating time spent in different activity behaviors in free living settings (16, 19, 20). These small non-invasive devices are also known to avoid self-report bias and are less expensive compared to doubly labelled water (21). Although accelerometers are relatively accurate in estimating activity in healthy adults (22), they have underestimated low-moderate intensity physical activity in people walking at a slower pace (23–27). Accelerometer data are also mostly interpreted by applying pre-determined thresholds to classify activity behavior intensities. These thresholds have been extensively validated in generally healthy populations, however the assumptions may be limited in CD, where ambulation is challenged, thus compromising the ability to make valid clinical recommendations on promoting healthy activity behaviors. The activPAL is commonly used in studies for the measurement of sedentary behavior (SB) and is considered accurate for this purpose (28) but may miscount steps in people living with chronic conditions that can cause them to walk slow (23–27, 29). Therefore, the aim of this study was to test the criterion validity of step counts and postural transitions detected with the activPAL accelerometer against direct observation in people living with cervical dystonia.

## 2 Methods

### 2.1 Study design

This was a single-group, single visit cross-sectional observational study where participants performed a series of functional tests in a laboratory setting.

### 2.2 Participants

Individuals with cervical dystonia over the age of 18 years were invited to take part in the study. Participants were recruited from the Neurology Department of two major hospitals in the North Island of New Zealand. Participants were also recruited via a dystonia-specific social media network in New Zealand. To be included in the study, participants must have been diagnosed with cervical dystonia of idiopathic origin, have had no other neurological or musculoskeletal condition affecting their ability to participate in physical activity (able to walk independently or with an assistive device), and have not received previous deep-brain stimulation procedures. Eligible participants were fully informed about the study and provided written consent prior to participating in the study. The Auckland Health Research Ethics Committee approved the study on 28/04/2020 (Reference Number AH1116).

### 2.3 Procedures

Participants performed all procedures at the Health and Rehabilitation Clinic at the Department of Exercise Sciences, University of Auckland, Auckland New Zealand.

The activPAL accelerometer (PAL Technologies, Glasgow, Scotland) was used in this study. The activPAL is a small lightweight triaxial inclinometer that uses static and dynamic accelerations and inclination of the thigh to determine the start and end of each period spent sitting/lying, standing, transitions and stepping (24, 30). The device was initialized and taped to the anterior mid-thigh of each participant’s preferred side of the body (right/left). The device was worn throughout a series of assessments and movement tasks.

Participants were asked to walk around a 50 m indoor track for 6 min at their usual walking pace. While the participant was walking, a researcher followed closely (2 m) behind and recorded step count by using a manual clicker (SHARP ELSI MATE EL-120 Tokyo, Japan). A step was considered from the heel strike of one foot to the heel strike of the other.

After the walking test, participants performed a 30-s sit-to-stand test as previously described (30). Participants were asked to stand from sitting on a box and repeat as many times as they could until the end of 30 s. The height of the box was determined from the participants height (box making around 90°^o^ angle at knees), ensuring a comfortable stand from sitting transition. The number of times the participant completed a transition was recorded. Hence, both measures, walking and sit-standing, were validated against direct observation, which is considered the gold standard while determining the counts of specific activity in the current study, step count and transitions (31).

#### 2.3.1 Data downloading and processing

Data was processed using the activPAL commercially available software (PAL software suite v8, PAL technologies Inc., Scotland). The “events extended” and “raw uncompressed acceleration” data files were generated using the default CREA algorithm (v 1.3). The CREA is considered more accurate than the VANE algorithm for transitions (32). There is an updated CREA version known as GHLA which includes calibration of the sensor. This algorithm is mainly for ActivPAL-4 plus and we used PAL-3 in our study. It is not known if this algorithm has any difference when catered to stepping and transitions in adults. Although it has been tested on children with results showing stepping mean absolute percentage of error being quite high for stepping (5–10 min) (33) these results although cannot be generalized on adults but it is suggested to make caution while comparing the results across studies using these algorithms. The start times of each of the activities (6-min walking and 30-s sit-to-stand) were synchronized to the same computer that the activPAL was initialized on, thus providing the same time stamp. The start and stop times in the activPAL datasets were identified and marked. The difference in cumulative step count at the start and end of the six-minute period was taken as the total number of steps taken in the 6 min. The total number of steps obtained from the activPAL for each individual was multiplied by 2 due to the activPAL recording reciprocal limb movements (leg backwards and forwards) as one-step, while direct observation recorded the leg backwards and then forwards as two steps. Walking cadence (number of steps per minute) was calculated by dividing the total number of steps by 6 min.

**Figure 1:**
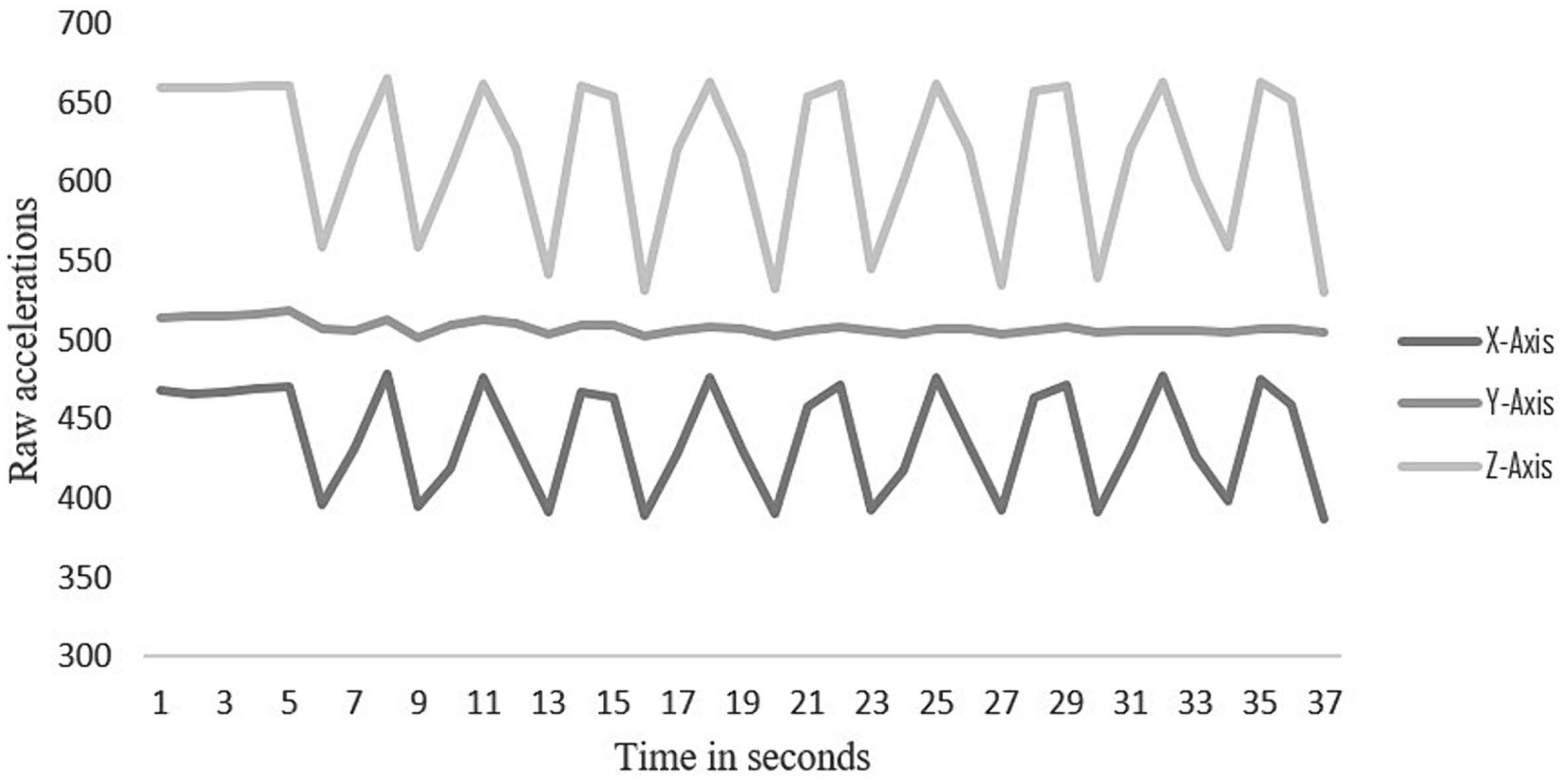
Raw data of one participant showing transitions between sitting and standing on the X, Y and Z axes.

The start and stop times of the 30-s sit-to-stand in the raw acceleration file were also identified and marked. The raw acceleration values along the x, y, and z-axes were graphed over the time-period and the number of peaks during that period were counted, one transition was considered from sitting to standing (Figure 1).

### 2.4 Statistical analysis

Data was analyzed using IBM SPSS V 23 and MS excel. Pearson’s correlation was used to determine the strength of the relationship between the outputs from the activPAL and those obtained through direct observation. The strength of the relationship was considered based on Dancey and Reidy (34) with ±1 representing perfect correlation, ±0.7 to ±0.9 strong, ±0.4 to ±0.6 moderate, ±0.1 to ±0.3 weak, and 0 considered as no relationship between variables. The Bland–Altman method was used to determine the agreement between the outputs obtained from the activPAL and those obtained through direct observation. The mean of the observations and the difference between the observations (activPAL and direct observations) were calculated and plotted. Statistical limits were calculated as mean ± 1.96 SD. In order for the two methods to have excellent agreement, 95% of the data points from the Bland–Altman plot were to lie within the ±1.96 SD (35). However, because of small sample we were able to collect, 90% agreement was acceptable based on Pedersen et al. (36) who considered 80% agreement acceptable.

## 3 Results

Twelve eligible individuals took part in the study and completed the procedures. Data from one participant for both assessments was not recorded on the activPAL due to an unknown technical error, while the sit-to-stand data from another participant was not synchronized. Therefore, data from 10 participants with valid sit-to-stand data and 11 participants with valid step count data were included in the final analysis. One participant used crutches as their mobility aid while performing the tests. All participants were using BOTOX^®^ as a treatment except for one participant, who stated the treatment was not effective for controlling their symptoms.

Participants (*n* = 6 female; *n* = 5 males) were older adults aged 61 ± 13 (mean ± SD) years with age range between 32 and 73 years. Participants had a BMI of 24 ± 3 kg/m^2^, ranges between 18 and 32 kg/m^2^ and the overall group had been living with dystonia for an average of 12 ± 7 years, the duration ranged between 3 and 20 years.

The number of steps and transitions obtained by direct observation and with the activPAL during the functional tests are presented in Supplementary Table S1.

**Figure 2:**
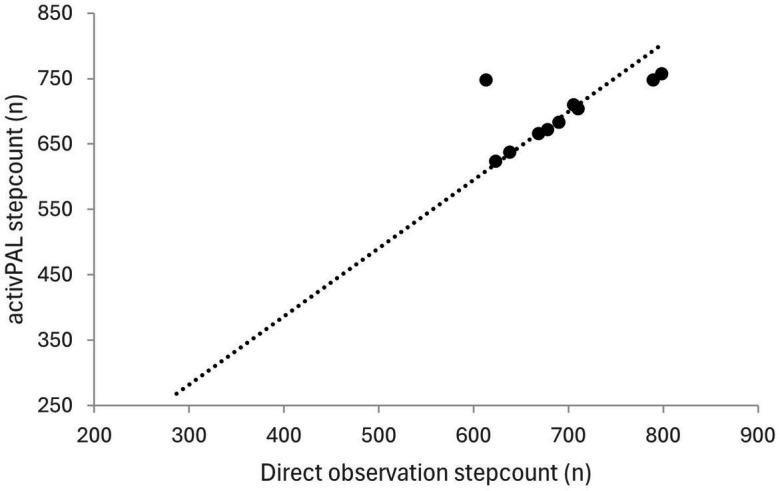
Correlation between step count measured with the activPAL and using direct observation (*r *= 0.941; *p* = 0.0001).

**Figure 3:**
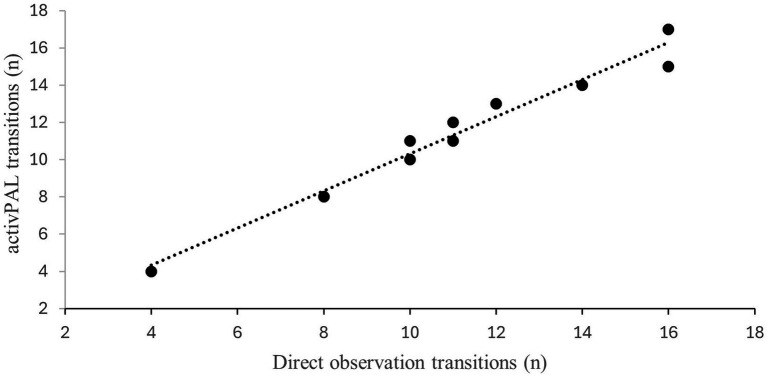
Correlation between transitions measured with the activPAL and using direct observation (*r *= 0.983; *p* =0.0001).

Both the number of steps and transitions between sitting and standing showed a strong positive correlation between the direct observation and activPAL using Pearson’s correlations. The strength of relation is presented as scatter dot plots for step counts during 6 min in Figure 2 and Transitions during 30 s in Figure 3 between the direct observation and activPAL (step count: *r* = 0.941, transitions: *r* = 0.983; *p* = 0.0001****).

**Figure 4:**
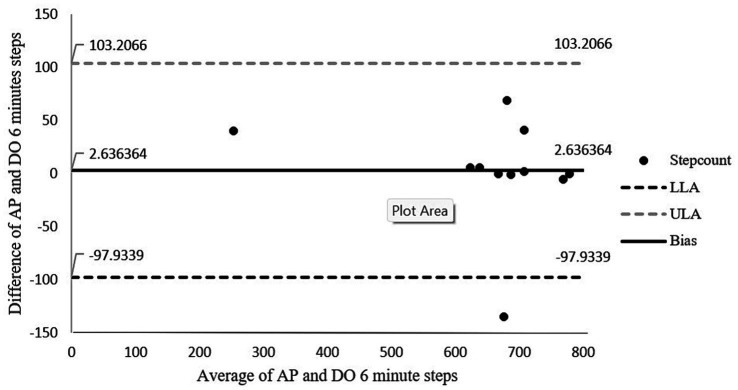
Bland-Altman plot for step count agreement between direct observation and the activPAL. The middle solid line represents the mean of difference (bias) while the outer dotted lines represent the limit of agreements. LLoA: lower limits of agreement; ULoA: upper limit for agreement; Bias: mean of the difference between the two methods.

Figure 4 shows the Bland–Altman plots for the number of steps taken during the 6 min of walking. There was 91% agreement between the number of steps of walking measured with the activPAL and direct observation. The absolute percentage of error for steps was 0.4%. The limits of agreements in this figure are wide and may be attributed to one of the outlier in the sample. As this outlier has a difference of 135 counts between direct observation and activPAL. The results did not change significantly even after removing the outlier. This outlier was in accordance with the inclusion criteria as well, hence there was no other reason for removing this outlier. Hence this analysis was done by adding the outlier.

**Figure 5:**
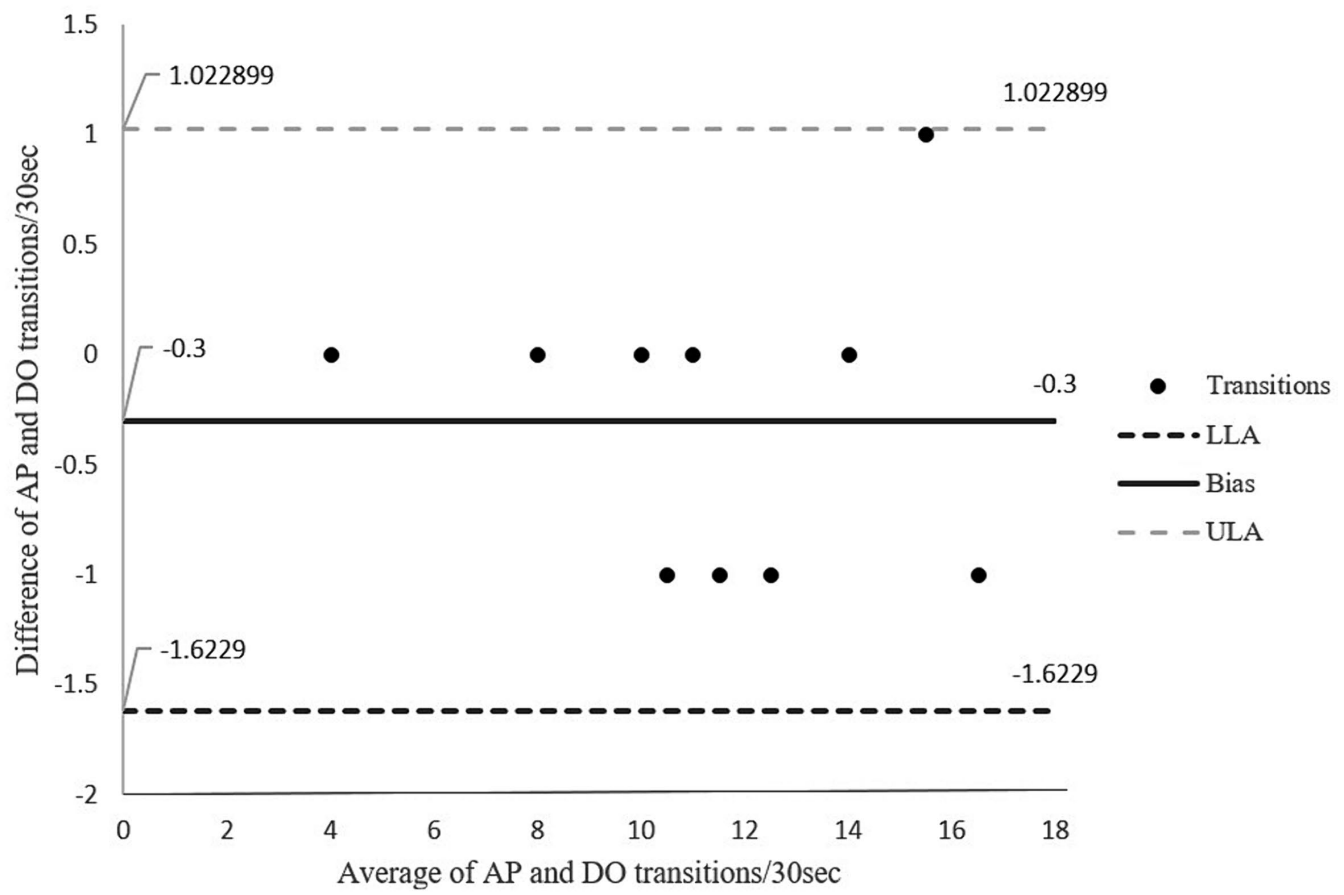
Bland-Altman Plot for agreement in transitions between sitting and standing for direct observation and activPAL transitions. The middle solid line represents the mean of difference (bias), the outer dotted lines represent the limit of agreements. LLoA: lower limits of agreement; ULoA: upper limit for agreement; Bias: mean of the difference between the two methods.

Figure 5 shows the Bland–Altman plot for the number of sit to stand transitions over a 30-s period. The graph shows good agreement of 100% between the two methods in which entire data lies within the limits of agreements. The absolute percentage of error for transitions was = 2.6.

## 4 Discussion

Overall, this study showed that the activPAL is a valid instrument to assess movement behavior in patients with CD. Our results showed a strong correlation between the criterion measures of directly observed step count and sit-to-stand transitions and those measured using the activPAL accelerometer. In addition, there was good to very high agreement between the measures of direct observation and the activPAL.

A previous study by Grant et al. (37) has used the activPAL to measure steps on a treadmill at five different speeds and during community walking at three self-selected speeds in healthy adults. The authors found that the activPAL was accurate in classifying the number of steps at all speeds including the lowest treadmill speed of 0.67 m/s where less than 100 steps/min were taken, and at the self-paced slow speed of 1.37 m/s (114 steps/min) (37). Furthermore, the absolute percentage error was <1% and there were narrow limits of agreements (37). The activPAL has been commonly used to measure sedentary behavior (SB) and is considered accurate for this purpose (28) but may miscount steps in people living with chronic conditions that can cause them to walk slow (23–27, 29). People with dystonia may present with balance problems, resulting in a slower walking pace (5). In our study, however there was high correlation and good agreement between direct observation and the activPAL for steps in our study. The usual walking speed of participants in our study was mostly at a cadence of >100 steps/min and may be a reason we demonstrated good agreement between measures. This walking cadence is considered as moderate intensity in adults (38–40). Participants in our study may have had normal function due to the reduced severity of their condition attributed to the fact that almost all participants were using BOTOX® as a treatment. In another study related to slow and fast walking in hospitalized patients, participants performed walking activities while wearing an activPAL and SENS monitor and while being directly observed (36). There was a moderate correlation between steps measured with the activPAL and direct observation at the two different speeds. Similarly, there was a good agreement (80%) between the activPAL and direct observation for steps at both walking speeds and the study reported an acceptable measure of error < 15% (36). Similar to the two aforementioned studies (36, 37), 91% of the data in our study was bound within ±1.96 SD, which is considered good agreement.

We also validated the number of transitions recorded by the activPAL against direct observation. A study that validated the activPAL against video recorded observation in healthy adults (41) determined that both step count and transitions were misclassified by the activPAL and the measurement methods showed poor agreement for the outputs in a lab as well as in free living setting (41). The authors reported the reason for the lack of agreement between methods for step count was that less purposeful and small strides were not detected due to the activPAL being attached to one thigh and the relatively long time taken for the activPAL to detect changes in posture. Similarly, in the study population of CD cohort, due to the lack of postural control at neck it is possible that the ActivPAL may have underreported some of the steps.

The number of transitions performed by participants in our study ranged between 4 and 16. A score of less than 12 is below the expected number of transitions for the age range of 60–64 and is indicative of a higher risk of falls (42). The low number in some participants is perhaps not wholly unexpected considering our participants had cervical dystonia. However, given that all our participants bar one appeared relatively functional based on their mean walking cadence (>100 steps/min), we would have expected participants to achieve a higher number of transitions. However repeated sit-to-stands assesses a different aspect of function to prolonged walking, and participants may have had low muscle endurance rather than slow walking ability. Nevertheless, our results showed a strong relationship and excellent agreement with narrow limits and a small margin of error (2.6%) between the two methods for transitions between sitting and standing.

### 4.1 Limitations

Although the activPAL was accurate in determining step count and transitions between sitting and standing in a controlled environment, we need to be cautious if extrapolating these data to the free-living setting. In addition, people having severe functional limitations may as well yield different results.

In addition, people having severe functional limitations due to dystonia may yield different results, and therefore a larger study on the timing of treatment and severity of disease impacts on activity behaviors should be conducted. Moreover, our study took place during the COVID-19 pandemic, and the unpredictability of the situation resulted in a small sample of participants. However, it is essential to highlight that cervical dystonia is a rare condition; therefore, we did not expect to gather a large sample size.

## 5 Conclusion

Based on the results of this study, the activPAL is a valid device to measure step counts and sit-to-stand transitions in individuals living with CD. The study found a good agreement between step counts and transitions measured with direct observation and that measured using activPAL.

## Data availability statement

The original contributions presented in the study are included in the article/Supplementary material, further inquiries can be directed to the corresponding authors.

## Ethics statement

The studies involving humans were approved by the Auckland Health Research Ethics Committee on 28/04/2020 (Reference Number AH1116). The studies were conducted in accordance with the local legislation and institutional requirements. The participants provided their written informed consent to participate in this study.

## Author contributions

IY: Conceptualization, Data curation, Formal analysis, Methodology, Writing – original draft, Writing – review & editing. SG: Conceptualization, Writing – review & editing. MS: Data curation, Writing – review & editing. RM: Conceptualization, Methodology, Supervision, Writing – review & editing.
