# Supplementary material for: Agreement between the activPAL accelerometer and direct observation during a series of gait and sit-to-stand tasks in people living with cervical dystonia
Source: Front Neurol. 2024 Apr 24;15:1286447. doi: 10.3389/fneur.2024.1286447 (PMC11080616; doi:10.3389/fneur.2024.1286447)
Supplement: Supplementary file 1 [file Table_1.docx]

Supplementary Material

# Supplementary Data

## Supplementary Table

Table 1 Number of steps and transitions recorded by activPAL and through direct observation.

| **Variable** | **DO** | **ActivPAL** |
| --- | --- | --- |
| **Total steps (n/6 min)** | 654 (287 - 798) | 651 (218 – 758) |
| **Cadence (steps/min)** | 109 (111-125) | 108 (48-131) |
| **Transitions (n/30 sec)** | 11 (4 - 17) | 11 (4 -16) |

*Data are mean (min-max). DO = direct observation.*
